# Automated Quantitative Analysis of p53, Cyclin D1, Ki67 and pERK Expression in Breast Carcinoma Does Not Differ from Expert Pathologist Scoring and Correlates with Clinico-Pathological Characteristics

**DOI:** 10.3390/cancers4030725

**Published:** 2012-07-18

**Authors:** Jamaica D. Cass, Sonal Varma, Andrew G. Day, Waheed Sangrar, Ashish B. Rajput, Leda H. Raptis, Jeremy Squire, Yolanda Madarnas, Sandip K. SenGupta, Bruce E. Elliott

**Affiliations:** 1 Division of Cancer Biology and Genetics, Cancer Research Institute, Queen’s University, Kingston K7L 3N6, Canada; E-Mails: 8jc22@queensu.ca (J.D.C.); ws4@queensu.ca (W.S.); raptisl@queensu.ca (L.H.R.); squirej@queensu.ca (J.S.); 2 Department of Pathology and Molecular Medicine, Queen’s University, Kingston K7L 3N6, Canada; E-Mails: varmas@kgh.kari.net (S.V.); drash3000@yahoo.com (A.B.R.); sengupts@kgh.kari.net (S.K.S.); 3 Kingston General Hospital, Kingston K7L 2V7, Canada; E-Mail: daya@kgh.kari.net; 4 Department of Oncology, Queen’s University, Kingston K7L 3N6, Canada; E-Mail: Yolanda.Madarnas@krcc.on.ca

**Keywords:** breast cancer, p53/cyclin D1/Ki67/pERK, tissue microarray, automated image analysis, clinico-pathological parameters

## Abstract

There is critical need for improved biomarker assessment platforms which integrate traditional pathological parameters (TNM stage, grade and ER/PR/HER2 status) with molecular profiling, to better define prognostic subgroups or systemic treatment response. One roadblock is the lack of semi-quantitative methods which reliably measure biomarker expression. Our study assesses reliability of automated immunohistochemistry (IHC) scoring compared to manual scoring of five selected biomarkers in a tissue microarray (TMA) of 63 human breast cancer cases, and correlates these markers with clinico-pathological data. TMA slides were scanned into an Ariol Imaging System, and histologic (H) scores (% positive tumor area x staining intensity 0–3) were calculated using trained algorithms. H scores for all five biomarkers concurred with pathologists’ scores, based on Pearson correlation coefficients (0.80–0.90) for continuous data and Kappa statistics (0.55–0.92) for positive vs. negative stain. Using continuous data, significant association of pERK expression with absence of LVI (*p* = 0.005) and lymph node negativity (*p* = 0.002) was observed. p53 over-expression, characteristic of dysfunctional p53 in cancer, and Ki67 were associated with high grade (*p* = 0.032 and 0.0007, respectively). Cyclin D1 correlated inversely with ER/PR/HER2-ve (triple negative) tumors (*p* = 0.0002). Thus automated quantitation of immunostaining concurs with pathologists’ scoring, and provides meaningful associations with clinico-pathological data.

## 1. Introduction

Basic discoveries in cancer biology over the past two decades have identified key signaling pathways that drive malignant progression in breast cancer, and panels of biomarkers that assess their activation [1]. Based on these studies, several commercially available molecular marker platforms (such as Oncotype Dx, Mammaprint) have been developed for use in certain types of clinical decision making [2]. However, there is a critical need for improved biomarker assessment platforms to integrate knowledge from traditional clinico-pathological variables such as tumor size and grade with pathway-based profiles that better define prognostic subgroups or systemic treatment response. One of the specific roadblocks in predictive oncology is the lack of accurate and reproducible assays based on molecular biomarkers for predicting therapeutic outcome or guiding patient selection during the early clinical stages of testing novel treatment modalities. A pathologist usually scores diagnostic immunohistochemistry (IHC) and tissue microarray (TMA) slides by bright field microscopy or occasionally by digitally scanned slides. Many factors can influence pathologists’ scoring, including varied ambient light conditions, amount of time scoring, fatigue and lack of standardization of routine stains [3]. We sought to determine if an objective, automated system, Ariol, could score a breast tissue microarray with the same accuracy as two pathologists. We also sought to determine if the automated quantification of our biomarkers of interest correlated with relevant clinico-pathological parameters.

Our main proteins of interest in this study were HER2, pERK, p53, cyclin D1, and Ki67, for which technical reliability of antibodies has previously been validated in IHC staining of tissue sections [4,5,6,7]. HER2 is amplified and over-expressed in approximately 15–20% of breast cancers, and is associated with increased recurrence and worse prognosis [8,9]. ERK, or Extra-cellular Regulated Kinase, is a member of the MAP kinase pathway, which can activate a variety of transcription factors that regulate cell proliferation. ERK is phosphorylated at Thr202/Tyr204 residues upon activation, and its phosphorylated form (pERK) is considered as a surrogate of cellular ERK activity. Aberrant over-expression of pERK expression frequently occurs in a variety of cancers [10], making the ERK pathway a potential target in cancer therapy [11].

Cyclin D1, p53 and Ki67 are regulators of cell cycle. Cyclin D1, a member of the cyclin-dependent kinase regulator family, acts as an activator of CDK 4 and CDK6 [12], and therefore as a positive regulator of cell proliferation. Aberrant amplification and over-expression of cyclin D1 is a driving force in 13–20% of human breast cancers, and is associated with poor disease outcome [13]. p53 is the most studied transcription factor involved in cancer and has been called “the Guardian of the Genome” [14]. p53 regulates genes involved in DNA repair and is a check point in cell cycle progression. p53 is mutated and over-expressed in approximately 25–30% of human breast cancers [15], with an increased incidence in triple negative (ER/PR/HER2-ve) breast cancers [16]. Ki67 is frequently used as a clinical measure of proliferation in tumors, and high Ki67 expression in combination with high p53 has been correlated with poor prognosis and treatment failures in breast cancer [17].

In the present study, we sought to assess concordance of visual and automated scoring methods for various biomarkers, and to explore associations of automated scores with established clinico-pathological parameters with the hope of providing a reference point for validation of automated quantitative scoring methods such as the Ariol imaging platform for use in clinical settings.

## 2. Results

### 2.1. Comparison of Manual *Versus* Automated Scoring

We observed a strong correlation between the manual and automated biomarker scores for the five biomarkers based on continuous data, ranging from 0.80 for p53 to 0.90 for HER2 (Table 1). When scores were categorized as positive or negative based on a threshold H score of >20, we found that chance corrected agreement between the two scoring methods ranged from Kappa = 0.55 for Ki67 to Kappa = 0.92 for pERK (Table 1). The proportion of tumors with positive biomarkers using Ariol scoring was: HER2 (25%), nuclear p53 (29%), cyclin D1 (65%), pERK (31%) and Ki67 (30%).

**Table 1:** Correlation of manual scoring and Ariol automated scoring of biomarkers.

Biomarker	Pearson Correlation Coefficient (95% CI)	Kappa Statistic (95% CI)	Proportion Positive ^+^
pERK	0.89 (0.75–0.97)	0.92 (0.80–1.00)	18/58 (31%)
p53	0.80 (0.65–0.92)	0.75 (0.56–0.95)	16/56 (29%)
Cyclin D1	0.85 (0.71–0.94)	0.73 (0.55–0.92)	37/57 (65%)
Ki67	0.81 (0.71–0.91)	0.55 (0.36–0.74)	17/56 (30%)
HER2	0.90 (0.83–0.95)	0.62 (0.40–0.84)	14/56 (25%)

CI, Confidence interval; ^+^: Determined based on threshold H score of >20. Denominators are less than 63 due to cores missing Ariol or manual scores.

### 2.2. Associations of Automated Scoring Between Biomarkers

We first correlated each of the biomarkers with one another using continuous scores. Of the ten pairs of correlations, none were significant (all *p* > 0.1 and r < 0.22), except p53 with Ki67 which had a correlation of 0.43 (95% CI, 0.05, 0.67) yielding a *p*-value of 0.0013 and a false discovery rate of 0.013. There was no significant association between any biomarkers using dichotomous scores (all Kappas < 0.25).

### 2.3. Associations of Biomarkers with Clinico-Pathological Parameters

**Figure 1:**
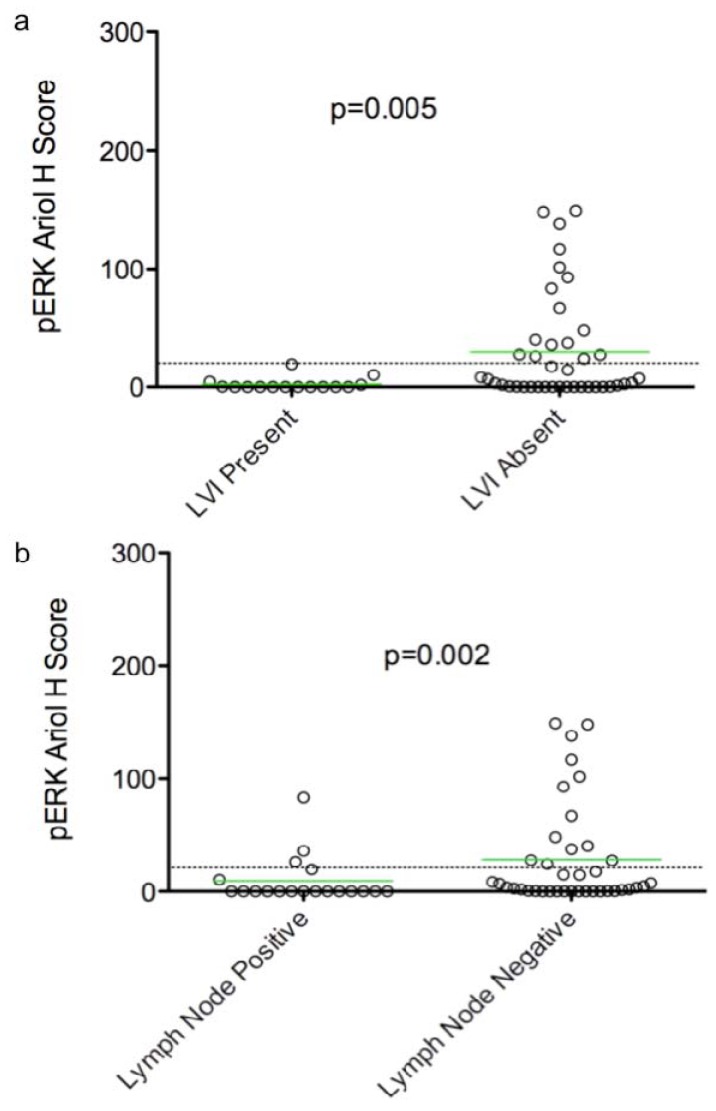
Dot plots of pERK Ariol H scores *versus* two clinico-pathological parameters. Dot plots of pERK Ariol H scores *versus* LVI (present, absent) (**a**) and lymph node (−,+) (**b**) status are shown. Significance between groups was determined using an exact Wilcoxon rank sum test, as described in Materials and Methods (*p* values indicated). Bars indicate the mean H score in each group, and the dotted line indicates the threshold for positive *versus* negative stain based on dichotomized data. Twenty five biomarker associations were tested in total. The dot plots displayed had a False Discovery Rate of <5% (see Experimental Section). The displayed *p*-values are unadjusted for the number of tests performed.

**Figure 2:**
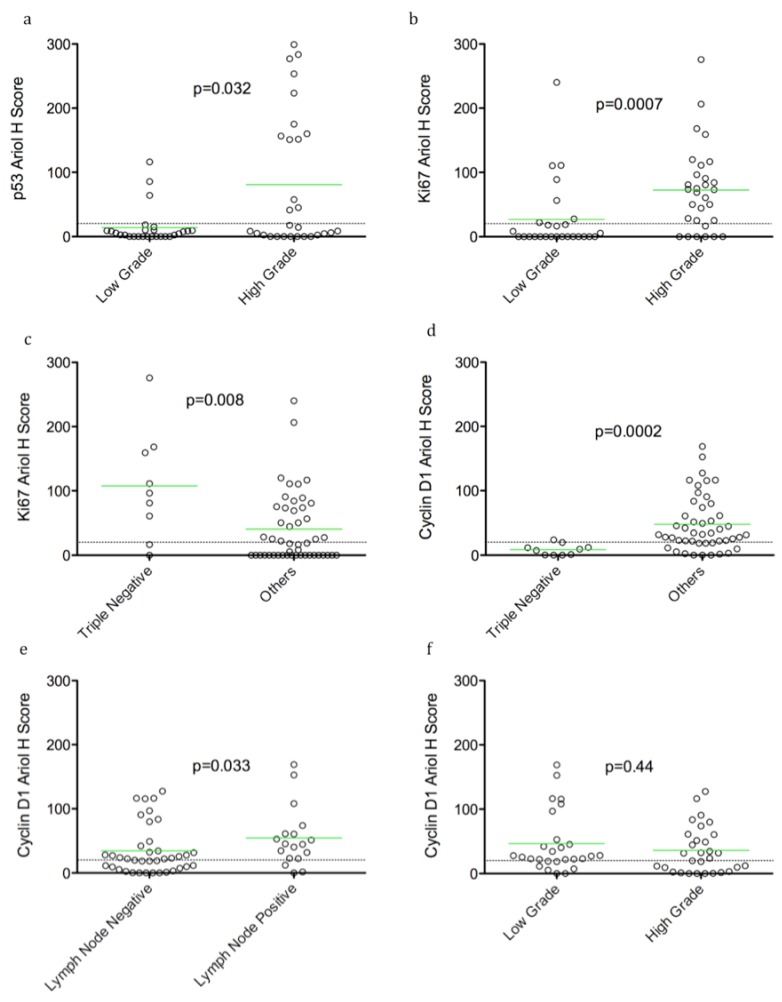
Selected dot plots of associations of p53, Ki67 and cyclin D1 with clinico-pathological parameters Dot plots of Ariol H scores of p53 (**a**), Ki67 (**b,c**) and cyclin D1 (**d–f**) *versus* selected clinico-pathological parameters are shown. Significance between groups was determined using an exact Wilcoxon rank sum test, as described in Materials and Methods (*p* value indicated). Bars indicate the mean H score in each group, and the dotted line indicates the threshold for positive *versus* negative stain based on dichotomized data. Statistical analysis was performed as in Figure 1. Examples of significant biomarker associations with indicated clinico-pathological parameters are shown (**a–e**). An example of no correlation of cyclin D1 with grade is shown for comparison (**f**).

In an exploratory analysis of continuous biomarker data, we found that over-expression of pERK was correlated with absence of LVI (*p* = 0.005) and lymph node negativity (*p* = 0.002) (Table 2, Figure 1). An association of p53 over-expression with high grade tumors was observed (*p* = 0.032). Ki67 positivity was also correlated with high grade (*p* = 0.0007), and inversely with triple negative cases (*p* = 0.008) (Table 2, Figure 2b,c). Thus p53 over-expression and Ki67 are associated with aggressive rapidly proliferating cancers. However, cyclin D1 expression correlated inversely with the triple negative tumor subset (*p* = 0.0002) (Table 2, Figure 2d), but showed no correlation with high grade (Table 2, Figure 2f). Consistent with its known adverse prognostic effect, a trend of HER2 association with recurrence (*p* = 0.096) was also evident (Table 2). Using dichotomized data (based on a threshold H score of >20), we observed a similar pattern of associations, except a correlation of pERK with lymph node negativity was not evident.

**Table 2:** Unadjusted bivariate association between biomarkers and clinico-pathologic parameters.

Biomarker (Ariol Score)	Clinical Parameter	n ^†^	Original continuous score			Score dichotomized as positive >20	
			Concordance index ^a^	*p*-value		Odds Ratio (exact 95% CI) ^b^	*p*-value
pERK	LVI (present)	56	0.25 ^c^	0.005 *^,c^		0.00 (0.00–0.41) ^c^	0.0028 *^,c^
	Lymph node status (+)	58	0.28 ^c^	0.002 *^,c^		NS	0.22
	SBR score ^d^ (8 or 9)	58	NS	0.37		NS	0.38
	ER/PR/HER2-ve (TN)	58	NS	0.89		NS	0.44
	Recurrence (yes)	51	NS	0.47		NS	1
p53	LVI (present)	54	NS	0.21		NS	0.74
	Lymph node status (+)	56	NS	1.00		NS	1.00
	SBR score (8 or 9)	56	0.67	0.032		6.5 (1.4–40)	0.0074 *
	ER/PR/HER2-ve (TN)	56	NS	0.11		NS	0.26
	Recurrence (yes)	48	NS	0.92		NS	1.00
cyclin D1	LVI (present)	55	NS	0.36		NS	1
	Lymph node status (+)	57	NS	0.033		4.3 (0.96–26.1)	0.041
	SBR score (8 or 9)	57	NS	0.44		NS	0.17
	ER/PR/HER2-ve (TN)	57	0.15 ^c^	0.0002 **^,c^		0.038 (0.001–0.34) ^c^	0.0003 **^,c^
	Recurrence (yes)	50	NS	0.69		NS	0.72
Ki67	LVI (present)	55	NS	0.42		NS	0.55
	Lymph node status (+)	57	NS	0.36		NS	0.57
	SBR score (8 or 9)	57	0.75	0.0007 **		9.4 (2.4–38)	0.0002 **
	ER/PR/HER2-ve (TN)	57	NS	0.008 *		NS	0.083
	Recurrence (yes)	50	NS	0.2		NS	0.15
HER2	LVI (present)	54	NS	0.16		NS	0.11
	Lymph node status (+)	56	NS	0.38		NS	0.76
	SBR score (8 or 9)	56	NS	0.13		NS	0.16
	ER/PR/HER2-ve (TN)	56	NS	0.24		NS	0.47
	Recurrence (yes)	49	0.65	0.096		NS	0.26

^a^ A concordance index <0.5 implies an inverse association while a concordance index >0.5 implies a direct association. Possible values range from zero (perfect discordance) to one (perfect concordance); ^b^ An odds ratio <1 implies an inverse association while and odds ratio >1 implies a direct association; ^c^ An inverse correlation was observed based on a and b above; ^d^ SBR score (8 or 9) denotes high grade tumours, compared to all others. * and ** denote false discovery rates of <0.05 and <0.01 accounting for the 25 comparisons.; Abbreviations: LVI, lymphovascular invasion; TN, triple negative; NS, not significant. **^†^** n = # of evaluable cases. Observations missing Ariol score or parameter do not contribute to the measures of bivariate association.

## 3. Discussion

In this study we have demonstrated strong concordance between manual and automated Ariol scoring for both dichotomized (positive *versus* negative) and continuous data for five extensively studied robust biomarkers. Both dichotomous and continuous scores yielded similar results with appropriate statistical testing, though the latter generally yielded a higher level of significance. Our findings indicate that our software algorithms have been properly optimized, and that Ariol analysis provides an objective means of automated quantification of IHC scoring. Automated Ariol methodologies are therefore reliable and may allow higher throughput, with standardized quantitative scoring for broader comparison among pathologists.

Although computer-assisted image analysis enables automated quantification of IHC staining intensity, its accuracy strongly depends on *a priori* lesion grading and epithelial/stromal compartment identification by trained Pathologists. Pathologic assessment is also crucial for selecting appropriate cut-offs for positive and negative stains, and for optimal training of algorithms. Our observed concordance between manual and automated scoring is similar to that reported previously for HER2 [18], estrogen/progesterone receptors [19,20] and aromatase [20]. However, the novelty of our study lies in the training of the Ariol computer algorithms to score the TMA slides. Moreover, we have created our own algorithms for both cyclin D1 and pERK and have shown that statistically they are as robust as the commercially available algorithms, and can yield relevant associations with clinico-pathological data. Furthermore, our study has extended Ariol-platform based analysis to include continuous as well as dichotomous scores for five biomarkers that could provide a more quantitative assessment for clinical correlative studies.

In an exploratory, hypothesis-generating analysis, automated Ariol scoring yielded some statistically significant correlations of specific pairs of biomarker and clinico-pathological parameters, using bivariate analysis. Furthermore, continuous and dichotomous (+ve *versus* –ve) data yielded similar results, except for pERK which correlated with lymph node negative status, Ki67 which correlated with triple negative cases, and HER2 which approached significance with recurrence using continuous but not dichotomous scores. Thus analysis of continuous data can validate thresholds set based on pathologists’ assessment and may provide improved statistical power for clinical correlative studies.

In this same cohort we have reported a significant increase in expression of Centromere Protein-A (CENPA) expression in invasive breast cancers compared to normal breast tissues using bivariate analysis of continuous data [21]. Similarly, a 50 case breast cancer study (CAN-NCIC-MA22) was used to demonstrate significant association of low tumor RNA integrity with response to chemotherapy [22]. While our study demonstrates the feasibility and potential reliability of this approach, the sample size is insufficient for multivariate analysis of biomarkers and clinical parameters. We believe this cohort is representative of an otherwise unselected population of premenopausal women with breast cancer given its assembly as consecutive premenopausal patients seen at a single institution over a defined timeframe. Whether our observations can be generalized to a population including postmenopausal women, or even male breast cancer, is unknown. Ultimately, validation of any biomarker correlations or associations with molecularly defined breast cancer subtypes and clinical outcome requires prospective validation of hypotheses so generated in a larger patient cohort with clinical follow-up data.

Several clinical studies have suggested that high pERK expression correlates with early stage node-negative breast cancer, and is an independent indicator of long relapse-free and overall survival [23]. Taken together, these studies indicate that ERK is not associated with enhanced *proliferation* and invasion of human breast carcinomas. Our analyses also show a correlation between pERK and LVI/lymph node negativity consistent with reported correlations between elevated pERK and early stage breast cancer. Other clinical studies however, show that ERK1/2 activity in primary tumors correlates with node-positivity, suggesting a correlation with late stage, metastatic breast cancer [24]. We speculate therefore that ERK activity may have different roles in early (initiation and progress) and late (metastatic) stages of tumor development. As a result, correlative relationships between pERK and clinical parameters and as well their “detectability” may be strongly dependent on tumor stage. Stratification of samples into early and late stage tumors may enhance the power and “detectability” of correlations, especially in studies on a larger cohort.

Previous reports have shown ERK regulates G1 cell cycle progression through activation of several immediate early genes, which in turn lead to induction of Cyclin D1, a major regulator of G1-S transitions [25]. Consistent with this, our data identify a correlation between pERK and proliferation (Ki67). However our data, as well as those of others, have not identified correlations between cyclin D1 and pERK and the reason for this is presently unclear [23]. We speculate that at early stages, ERK activity is sensitized to regulation by stromal influences (that include growth-factors and ECM), and hence it may exhibit temporally *transient* fluctuations in its steady-state activity. Thus the window of detection may be small and would hamper detection of correlations with cyclin D1, especially in the reduced sample size of our representative cohort. Moreover, signal regulatory mechanisms are more likely to be intact in the early stages of breast cancer. Hence, pERK signal may be immediately down-regulated upon cyclin D1 induction by feedback mechanisms. This would further reduce the window of detection for correlations [25]. Lastly, since ERK activity associated with upregulation of cyclin D1 requires ERK translocation to the nucleus, we examined nuclear pERK activity to optimize unmasking of correlations in our study. However, correlations masked by feedback dependent down-regulation of ERK activity (post-cyclin D1 induction) could be detected if nuclear localization of *inactive *ERK was used as a surrogate marker of cyclin D1 transcriptional induction. In this regard it is interesting that correlations between cyclin D1 and inactive (nonphosphorylated) ERK have been reported [23].

We detected positive correlations between TN tumors and proliferation (Ki67 staining). Surprisingly, however an inverse correlation between TN tumours and cyclin D1 levels was found. This finding is consistent with previously reported associations of cyclin D1 with better prognosis in breast cancer [26,27,28]. However, in addition to their role in promoting cell cycle entry, evidence suggests that cyclin D1 over-expression also serves to maintain proliferation and concomitantly inhibit differentiation [25]. We speculate that cyclin D1 levels may be reduced in advanced terminally-differentiated metastatic tumors, as cells at this stage no longer require cyclin D1’s regulatory effects on proliferation and differentiation. Indeed these cells may have acquired terminal invasive states in which upstream inputs are uncoupled from cyclin D1 induction. Such cells may take constitutive proliferative and differentiative cues instead, from aberrantly functioning downstream components such as Rb and E2F [29]. Hence reduced cyclin D1 levels may be an important marker for TN tumors and warrants additional confirmation in a larger cohort.

## 4. Experimental Section

### 4.1. Patients

With Queen’s University Research Ethics Board approval, breast tumor specimens were collected from 63 consecutive consenting female patients who received treatment for breast cancer at the Cancer Centre of Southeastern Ontario at Kingston General Hospital between 2005 and 2007. Clinico-pathological information for each case was retrospectively obtained from the electronic and paper patient record and entered into an anonymized database by an experienced oncologist. Archival normal breast tissues from twenty reduction mammoplasty specimens were included to provide non-malignant controls. Patients included in the study were premenopausal (less than 49 years of age at diagnosis), had primary invasive mammary carcinomas (>90% are ductal and/or lobular) and were stage T1-3a, N0-1, M0. Patients were excluded if they had any previous history of cancer, bilateral breast disease or neoadjuvant chemotherapy. Mean age of this patient cohort was 43.5 years, (range 29–49). The majority of the patients (60%) had N0 disease and received adjuvant chemotherapy (74%). Tumor grade was defined, based on tubule formation, mitotic activity and nuclear size, and showed the following distribution based on SBR (Scarff-Bloom-Richardson) score: grade I (SBR 3–5, 14%) grade II (SBR 6–7, 37%) and grade III (SBR 8–9, 51%). ER, PR and HER2 receptor status of the patient cohort, based on immunohistochemistry, defined a subgroup (14%) of triple negative (ER/PR/HER2-ve) breast cancers in the cohort (Table 3). As the cohort was assembled from consecutive consenting patients, there was no selection bias for any prognostic variables tested. Survival was defined as the number of patients that were alive or had recurrence up to the summer of 2010.

**Table 3:** Clinico-pathologic characteristics of patients included in the study (63 tumor cohort).

Parameter	Status	Number (%)
Age	<30	1 (2.1)
(Median: 45)	30–40	11 (22.9)
(Range: 29–49)	41–49	36 (75)
Tumor Stage	stage 1	26 (54.2)
	stage 2	16 (33.3)
	stage 3	1 (2.1)
	stage 4	1 (2.1)
	Unknown	4 (8.3)
Tumor Grade ^a^	Grade I	8 (12.7)
	Grade II	23 (36.5)
	Grade III	32 (50.8)
LVI	Absent	42 (64.3)
	Present	15 (35.7)
Number of positive lymph nodes	0	21 (60)
	1–3	11 (31.4)
	4–10	1 (2.9)
	>10	2 (5.7)
ER Status	Negative	14 (29.2)
	Positive	34 (70.8)
PR Status	Negative	12 (25)
	Positive	36 (75)
HER2 Status ^b^	Negative	36 (75)
	Positive	9 (18.8)
	Missing value	3 (6.2)
ER/PR/HER2 Status	Triple negative	10 (14)
	Others	53 (86)
Survival	Positive	11 (17)
	Negative	43 (68)
	Missing value	9 (15)

^a^ Tumor grade is determined based on SBR score (See Experimental Section); ^b^ HER2 staining was scored using the Hercept test^®^ scoring system (See Experimental Section).

### 4.2. Tissue Microarray Construction

Primary breast cancer specimens were routinely formalin fixed and paraffin embedded (FFPE) in the Queen’s Laboratory of Molecular Pathology (QLMP) and Kingston General Hospital. From this material, we constructed primary breast cancer TMAs in the QLMP. Sections of FFPE primary tumors were first stained with hematoxylin and eosin and reviewed by a pathologist. Representative tumor areas were circled and matched with the donor blocks. From each donor block, three 0.6-mm cores were punched out and embedded 1 mm apart in a recipient block using a Tissue Microarrayer (Beecher instruments, Silver Springs, MD, USA). A technical TMA for antibody optimization was constructed consisting of 8 breast tumors and 4 normal breast tissues from reduction mammoplasty specimens. Two test TMAs consisting of tissues from our 63 tumor cohort and 20 normal mammoplasty specimens were used for correlational studies.

### 4.3. Immunohistochemistry (IHC)

IHC was performed on 5 μm thick TMA sections for pERK (#4370, Cell Signaling, Boston, MA, USA), p53 (#760-2542, Ventana Medical Systems, Tuscon, AZ, USA), Ki67 (#790-4286, Ventana Medical Systems) and cyclin D1 (cat# RM-9104-S, Neo Markers, Freemont, CA, USA), according to REMARK guidelines [30]. Antigen retrieval was done with citrate buffer (pH 6.5) and slides were stained manually overnight at 1:100 dilution (for cyclin D1) or using the Ventana Benchmark automated staining system (Ventana Medical Systems, Tucson, AZ, USA) (for p53 and Ki67). Normal tonsil tissue was used as positive control for cyclin D1, Ki67, and p53. The pERK antibody used in our study has previously been used for staining of breast tumor tissues [23,31] and was optimized manually (citrate buffer, pH 6.5), and then for Ventana staining (1/200 dilution) using protocol #82 CC. HER2+ve breast tumor *versus* normal breast tissues were used as positive and negative controls. In all clinical cases, we routinely assessed ER/PR staining (see below) in normal ducts *versus* tumor regions from whole sections, as an internal control for tissue quality (e.g., normal ducts should show focal immunoreactivity of ER/PR). Technical reproducibility was tested for each biomarker by comparing replicate staining of serial sections from whole tissue blocks or the technical 8 tumor TMA. We looked at the overall intensity and gradations in the staining while comparing the cancer cells and interspersed stromal elements. Although there were minor differences between two consecutive sections, the overall staining intensity and pattern of staining was almost identical (data not shown). Tumor heterogeneity was assessed by comparing stained sections from each of two test TMAs for cyclin D1, p53, and pERK. The two TMAs represent three cores each from different areas of the same tumor, thus allowing us to assess tumor heterogeneity. Excellent reproducibility was observed between H scores for each marker from the two TMAs, as determined by Pearson/Spearman correlations (0.79–0.82), indicating minimal intra-tumor heterogeneity of expression for our biomarkers. The slides were also stained for ER, PR and HER2 (Clone 4B5) on the Ventana system using the respective Ventana antibody kits (pre-diluted by supplier—Ventana).

### 4.4. Manual Scoring

For pERK, p53, cyclin D1 and Ki67 staining, the % positive tumor area and nuclear staining intensity (scale of 0–3) were scored by two pathologists independently, with resolution of discordant cases by a senior pathologist. Cores that were lost/damaged during sectioning or had less than 10% of tissue with tumor were not scored, and the number of evaluable cases for each analysis is indicated in Table 1 and Table 2. A histo (H) score was then calculated for each core by multiplying % positive area and staining intensity for a value from 0–300, and expressed as the average of 3 cores per tumor. For ER and PR staining, the fractions of positive tumor nuclei were scored as 0 (<1%), 1+ (1–25%), 2+ (25–75%), and 3+ (>75%). The data for ER/PR staining were dichotomized into negative (0) *versus* positive (>1+) cases. HER2 membranous staining was scored using the Hercept test^®^ (Dako Corporation, Carpinteria, CA, USA) scoring system as “0” (no staining or membrane staining in <10% of the tumor cells); “1+” (incomplete membrane staining in >10% tumor cells); “2+” (weak to moderate complete membrane staining in >10% of tumor cells); “3+” (strong complete membrane staining in >10% of tumor cells). The data for HER2 staining were categorized into negative (<1+) *versus* positive (>3+) cases. In this study, breast cancer cases were tested for HER2 in the era prior to the ASCO/CAP guidelines (2007) requiring 30% of invasive carcinoma cells showing 3+ membrane staining [32] and patient care decisions were made upon the basis of those results. The incidence of HER2 overexpression for these cases was 18% (Table 3)—within the range reported in the literature. These values along with ER/PR status, were therefore used to define triple negative cases in this study.

### 4.5. Automated Scoring

**Figure 3:**
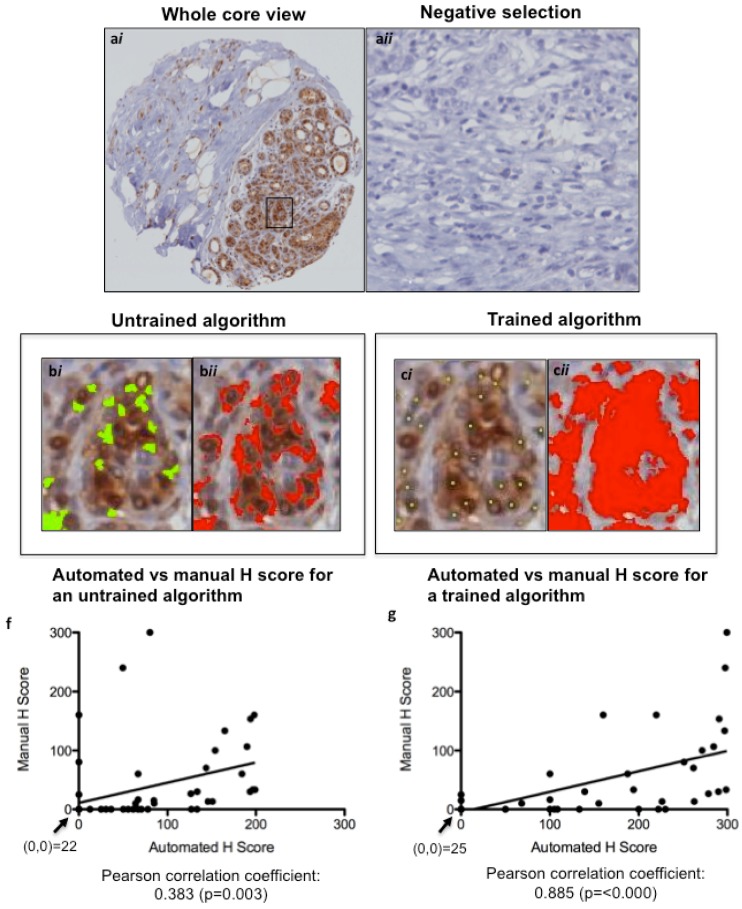
Optimizing Ariol Software for pERK IHC scoring. A TMA slide immunostained for pERK was scanned into the Ariol Sl-50 slide scanner (**a**) and a nuclear analysis was done without (**b**) or with (**c**) training based on size/shape characteristics (**b*i***, **b*ii***) and color (**c*i***, **c*ii***). The same cores were scored manually by two pathologists. The untrained (**d**) and trained (**e**) automated H scores were each plotted against the “gold standard” manual H Scores, and a Pearson correlation coefficient (with *p* value) was calculated. A linear regression line of best fit is shown. The values at the origin in each plot are indicated. (**a**), 200× magnification; (**b** and **c**), 600× magnification.

**Figure 4:**
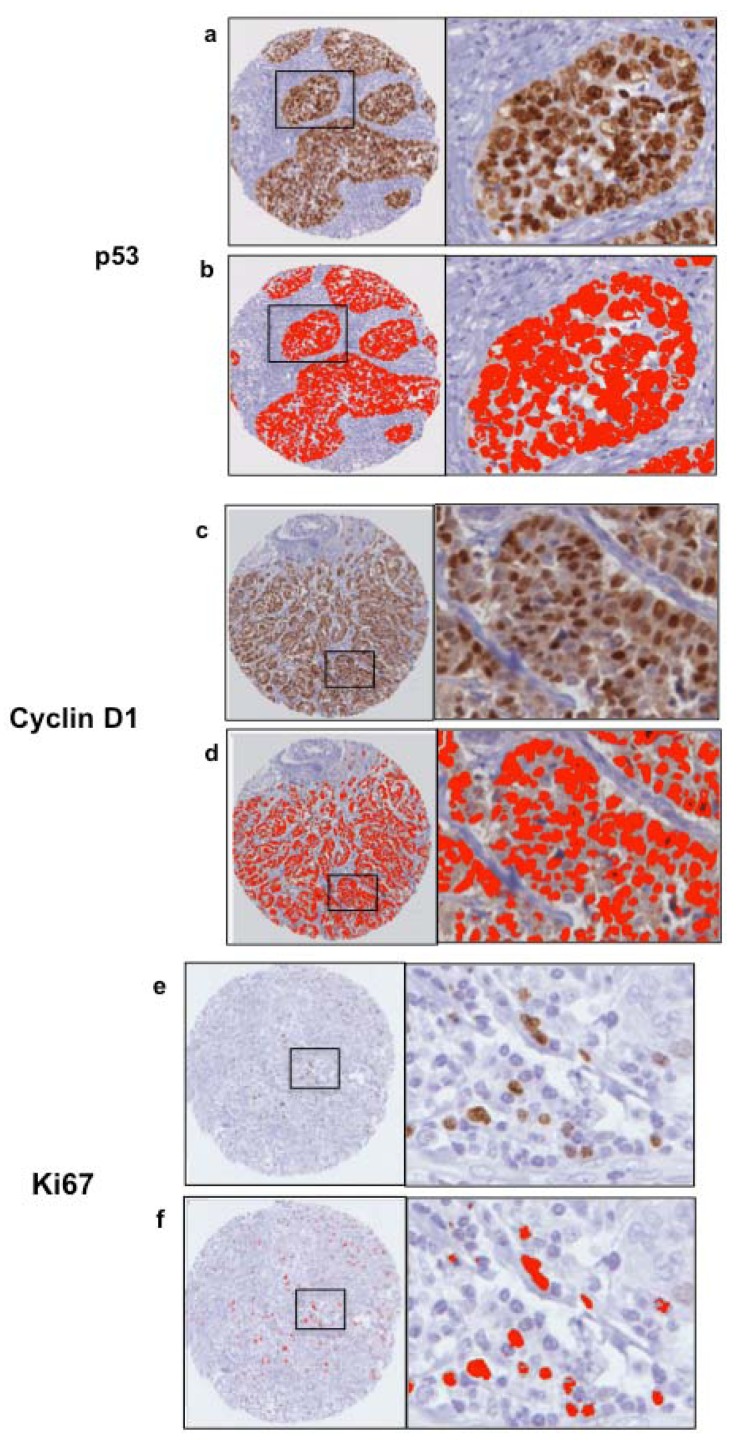
Gating of p53, cyclin D1 and Ki67 staining using trained Ariol algorithms. Examples of positive immunostaining for p53, cyclin D1 and Ki67 are shown (**a,c,e**). Optimized Ariol color classifiers are shown as a red overlay (**b,d,f**). 100× magnification, left, and 600× magnification, right.

TMA slides were scanned into the Ariol Image Analysis System SL-50 (Leica, San Jose, CA, USA), and an image analysis protocol was adapted based on previous studies for HER2 [18,19]. Scoring of algorithms was optimized using a nuclear script, which gates all hematoxylin-stained tumor nuclei based on geometric characteristics such as size, shape, compactness and roundness. This allows for scoring only of tumor area, ignoring stromal components such as fibroblasts and tumor-infiltrating lymphocytes. Positive tumor nuclei are gated on color, hue and intensity of brown staining (shown for pERK in Figure 3a), as well as geometric characteristics. This allows for calculation of percentage positivity on a cell-by-cell basis. The script is optimized on training areas from several cores and multiple patients (Figure 3b,c). The untrained and trained automated H scores were each plotted against the manual H Scores, and a Pearson correlation coefficient (with *p* value) was calculated (Figure 3d,e) to assess concordance. For p53 and Ki67, commercially available baseline scripts were optimized for our staining, while for cyclin D1 and pERK a generic nuclear script from the company software was optimized for scoring (Figure 3 and Figure 4). A conversion formula for the staining intensity provided by the manufacturer was used in the calculation of H scores, analogous to the calculation used for manual scoring.

### 4.6. Statistical Analysis

Two types of analyses were done, using (a) binarized data (scored +ve or –ve), and (b) continuous data (no threshold). The choice of cut-point for binarized data was somewhat arbitrary, but was based on the distribution of the markers rather than optimizing the test/agreement performance. We noted that for most of the markers, the values were either near zero or quite a bit greater than 20, so we considered values less than 20 as negative since these values likely differ from zero only by noise due to the limited accuracy of the method. Using the data to choose “optimal cut-points” for each marker is to be avoided with such a small sample size, as this approach would greatly overestimate the performance of the markers and could introduce additional bias. Therefore, we dichotomized the Ariol and manual scores for all biomarkers at 20 and considered values >20 as positive and <20 as negative.

Pearson’s correlation coefficient was used to measure the correlation between the Ariol and manual continuous scores, as well as the correlation between the various Ariol biomarker scores. Since the scores were not normally distributed we used the non-parametric percentile based bootstrap with 10,000 replications to estimate confidence intervals for the correlation coefficients. The agreement between scoring methods and associations among dichotomized biomarker scores is described by Cohen’s Kappa statistic which corrects for expected chance agreement.

For testing associations of biomarkers with clinico-pathological parameters two types of analyses were done, using (a) binarized (scored +ve or –ve) or (b) continuous (no threshold) Ariol scores. Associations of binarized biomarker scores with clinico-pathological parameters were determined by Fisher exact test. For associations of continuous biomarker scores with clinico-pathological parameters, we used the exact Wilcoxin rank-sum test, which assesses whether one of any two samples of independent observations tend to have larger values than the other.

Next, we assessed the association between the dichotomized Ariol biomarker scores and: grade, LVI, lymph node status, ER/PR/HER2 status and recurrence. Grade was dichotomized into low (I + II) and high (III); ER/PR/HER2 receptor status was dichotomized as triple negative *versus* all other subtypes (Table 3). The continuous Ariol scores were compared between the dichotomized clinico-pathological variables by the exact Wilcoxon-rank-sum test. The association between clinico-pathological variables and Ariol is described by the concordance index which is the probability that someone with a positive clinico-pathological variable has a higher Ariol score than someone with a negative clinico-pathological variable plus half the probability that they have the same Ariol score. The concordance index is also known as the *C*-statistic which is equivalent to the area under the Receiver Operating Characteristics curve [33]. The strength of association between “positive” (*i.e*., >20) Ariol biomarker values and the clinico-pathological variables are described by odds ratios with exact 95% confidence intervals and tested by Fisher’s exact test. A concordance index of <0.5 or odds ratio of <1 implies an inverse correlation, while a concordance index of >0.5 or an odds ratio of >1 implies a direct correlation. We report unadjusted p-values, but to account for the large number of tests we note comparisons that have false discovery rates below 5% and 1% [34]. The analysis was conducted using SAS version 9.1 (SAS Institute Inc., Cary, NC, USA).

## 5. Conclusions

In this paper, we have applied an improved automated method for quantifying biomarker expression in human breast cancer cases, using several robust biomarkers that have clinical relevance. Concordance between manual and automated scoring may assist researchers in more efficient quantitative analysis of TMAs with larger patient cohorts, and in discovery of novel prognostic/predictive biomarkers. Furthermore, analysis of continuous data validated results obtained using dichotomous scores, and provided enhanced statistical power. Whereas our observed biomarker correlations with specific clinico-pathological variables reflect previous reports in the literature, further validation in a larger dataset is required. Moreover, the implication of larger scale biomarker evaluations for crucial management decisions requires that these reproducible automated methods be introduced into clinical laboratories over the next several years.
